# Combination of Oncolytic Measles Virus and Ursolic Acid Synergistically Induces Oncolysis of Hepatocellular Carcinoma Cells

**DOI:** 10.3390/v15061294

**Published:** 2023-05-31

**Authors:** Ching-Hsuan Liu, Chen-Jei Tai, Yu-Ting Kuo, Shen-Shong Chang, Liang-Tzung Lin

**Affiliations:** 1 Department of Microbiology and Immunology, School of Medicine, College of Medicine, Taipei Medical University, Taipei 110, Taiwan; 2 Department of Microbiology & Immunology, Dalhousie University, Halifax, NS B3H 4R2, Canada; 3 Department of Obstetrics and Gynecology, School of Medicine, College of Medicine, Taipei Medical University, Taipei 110, Taiwan; 4 Ph.D. Program in Clinical Drug Development of Herbal Medicine, Taipei Medical University, Taipei 100, Taiwan; 5 Department of Medical Imaging, Chi Mei Medical Center, Tainan 710, Taiwan; 6 Division of Gastroenterology, Taipei City Hospital Yang-Ming Branch, Taipei 111, Taiwan; 7 Department of Medicine, School of Medicine, National Yang Ming Chiao Tung University, Taipei 112, Taiwan; 8 Institute of Public Health, National Yang Ming Chiao Tung University, Taipei 112, Taiwan; 9 Graduate Institute of Medical Sciences, College of Medicine, Taipei Medical University, Taipei 110, Taiwan

**Keywords:** hepatocellular carcinoma, oncolytic virotherapy, measles virus, ursolic acid, drug combination, hepatitis B virus, hepatitis C virus

## Abstract

Hepatocellular carcinoma (HCC) remains a difficult-to-treat cancer due to late diagnosis and limited curative treatment options. Developing more effective therapeutic strategies is essential for the management of HCC. Oncolytic virotherapy is a novel treatment modality for cancers, and its combination with small molecules merits further exploration. In this study, we combined oncolytic measles virus (MV) with the natural triterpenoid compound ursolic acid (UA) and evaluated their combination effect against HCC cells, including those harboring hepatitis B virus (HBV) or hepatitis C virus (HCV) replication. We found that the combination of MV and UA synergistically induced more cell death in Huh-7 HCC cells through enhanced apoptosis. In addition, increased oxidative stress and loss of mitochondrial potential were observed in the treated cells, indicating dysregulation of the mitochondria-dependent pathway. Similar synergistic cytotoxic effects were also found in HCC cells harboring HBV or HCV genomes. These findings underscore the potential of oncolytic MV and UA combination for further development as a treatment strategy for HCC.

## 1. Introduction

Liver cancer is one of the leading causes of cancer death worldwide, and hepatocellular carcinoma (HCC) is the most common type of primary liver cancer [1]. HCC is a major health concern due to its high and increasing incidence rate, poor prognosis, and limited treatment options [2]. The number of new cases and deaths from liver cancer are estimated to increase by more than 55% by 2040 if the current rates do not change [3]. The major risk factors of HCC include chronic hepatitis B virus (HBV) and hepatitis C virus (HCV) infections, aflatoxin-contaminated foods, chronic alcohol consumption, non-alcoholic fatty liver disease, excess body weight, type 2 diabetes, smoking, and cirrhosis from any other etiologies [1,2]. Treatment options for HCC depend on the stage and extent of the tumor, as well as the underlying liver function. Surgical resection, liver transplantation, and local thermal ablation therapies are considered potentially curative treatments for early-stage HCC [4]. For advanced stages, systemic therapies with sorafenib or lenvatinib are the first-line treatment [4]. Novel targeted agents regorafenib, cabozantinib, and ramucirumab are recently approved by the U.S. Food and Drug Administration (FDA) and European Medicines Agency (EMA) as second-line therapies based on promising clinical trial results [2,5]. Immunotherapies including nivolumab, pembrolizumab, and nivolumab plus ipilimumab have also been approved by the U.S. FDA [2]. Nonetheless, more effective treatment strategies are required to improve the prognosis of HCC.

Oncolytic virotherapy is another promising treatment strategy for HCC. Several studies have shown that oncolytic viruses (OVs) can selectively infect and replicate in HCC cells, leading to cell death and tumor regression [6]. Examples include adenovirus, herpes simplex virus, vaccinia virus, vesicular stomatitis virus, reovirus, influenza virus, Newcastle disease virus, and alphavirus M1 [6]. Engineered measles virus (MV) has also shown anti-HCC activity in preclinical studies [7,8,9]. However, further studies are needed to optimize the MV as an oncolytic vector and determine its potential as a treatment for HCC. For instance, combination of MV with another anti-cancer drug may be a feasible approach, as a previous study demonstrated the synergistic effect of MV and oral histone deacetylase (HDAC) inhibitor resminostat on HCC cells [10].

Natural phytochemicals have started to be investigated as agents to boost oncolytic virotherapy due to their diverse anti-cancer effects that often synergize with virus-induced tumor oncolysis. Such synergistic combination not only permits the achievement of significant anti-cancer effect at low doses of the OV and phytochemicals, hence reducing cytotoxicity compared to using each agent alone, it can also help overcome resistance problems that arise from monotherapy. Examples include baicalein, cinnamaldehyde [11], and ursolic acid (UA) [12], that were previously observed to boost MV oncolytic activity against breast cancer cells. UA, a natural triterpenoid compound found in various fruits and herbs, has been shown to possess multiple anti-cancer effects including against HCC [13]. Several preclinical studies have demonstrated that UA can inhibit HCC cell proliferation, induce cell cycle arrest and apoptosis, and suppress tumor growth in vitro and in vivo [14,15,16,17,18,19,20]. Furthermore, UA has been shown to have synergistic effects when combined with other anti-cancer agents, such as sorafenib, a first-line treatment for HCC. This combination therapy was found to enhance the anti-cancer effects of both drugs [21]. Overall, UA has great potential as a natural compound for combination treatment of HCC.

Given the applicability of both oncolytic MV and UA in HCC and potential benefits of drug combination, we explored UA’s potential application as an enhancer to MV-induced oncolysis in HCC. We evaluated the synergistic effect of the MV and UA combination and investigated the underlying mechanisms contributing to the enhanced cell death. HCC cells harboring HBV or HCV replicating genomes were further examined for their susceptibility to this combination.

## 2. Materials and Methods

### 2.1. Cell Culture, Virus, and Reagents

Human HCC cell line Huh-7 [22] (kindly provided by Dr. Stanley M. Lemon, University of North Carolina at Chapel Hill, USA) were cultured in Dulbecco’s Modified Eagle Medium (DMEM; Gibco™, Thermo Fisher Scientific; Waltham, MA, USA) containing 10% fetal bovine serum (FBS; Gibco™), 50 µg/mL of gentamicin (Gibco™), and 0.25 µg/mL of amphotericin B (Gibco™) in a humidified 5% CO_2_ incubator at 37 °C. HepG2.2.15 cells (kindly provided by Dr. D. Lorne J. Tyrrell, University of Alberta, Canada) were maintained in DMEM/F-12 (Gibco™) containing 10% FBS, 50 µg/mL of gentamicin, 0.25 µg/mL of amphotericin B, and 200 μg/mL of G418 (GeneTeks Biosciences; New Taipei City, Taiwan). Huh-7 derivatives harboring replicating HCV RNA (Huh-7.HCV1cc, Huh-7.H77Ccc, and Huh-7.Jc1 cells) were generated by electroporating full-length in vitro transcribed genomes from genotype 1a HCV (pHCV1cc and pH77Ccc [23]; kindly provided by Dr. Jens Bukh, University of Copenhagen, Denmark) and genotype 2a (Jc1FLAG2(p7-nsGluc2A) [24]; kindly provided by Dr. Charles M. Rice, Rockefeller University, USA) constructs as previously described. The recombinant Ichinose-B 323 strain measles virus tagged with enhanced green fluorescent protein (MV-EGFP) or luciferase (MV-Fluc) (kindly provided by Dr. Christopher D. Richardson, Dalhousie University, Canada) was propagated in marmoset B lymphoblastoid B95a cells as previously reported [11]. Viral titer was determined using the 50% tissue culture infective dose (TCID_50_) assay, and virus concentrations were represented as multiplicity of infection (MOI). Ursolic acid (UA) was purchased from Sigma-Aldrich Chemicals Co. (St. Louis, MO, USA). All other experimental reagents used were of analytical grade.

### 2.2. Cell Viability Assay

Cell viability was assessed using the Cell Counting Kit-8 (CCK-8; Sigma-Aldrich). Cells seeded in 96-well plates (10^4^ cells per well) were treated with MV or UA for the indicated incubation time. Cell viability was then determined following the manufacturer’s instructions and calculated with the equation Cell viability (%) = (Absorbance_test compound_/Absorbance_control_) × 100%. The 50% cytotoxic concentrations (CC_50_) of MV and UA were determined using the [Inhibitor] vs. Normalized response—Variable slope equation with least squares fit in GraphPad Prism software (version 9.0.2; GraphPad Software; San Diego, CA, USA).

### 2.3. Evaluation of Synergistic Effect

To determine the synergistic effect of MV and UA combination, cells seeded in 96-well plates (10^4^ cells per well) were infected with MV for 1.5 h, washed with phosphate-buffered saline (PBS; HyClone, GE Healthcare, Chicago, IL, USA), and incubated for 48 h, and then treated with UA for another 72 h. Cell viability was measured with the CCK-8 assay as described above. The combination effect was determined using CompuSyn software developed by T. C. Chou and Nick Martin [25] based on the Chou–Talalay method, in which the combination index (CI) indicates additive (CI = 1), synergistic (CI < 1), or antagonistic (CI > 1) effect [26].

### 2.4. Western Blot Analysis

Cells seeded in 12-well plates (2 × 10^5^ cells per well) were infected with MV-EGFP (MOI 0.1), incubated for 48 h, and treated with UA (75 µM) for another 72 h. Cell lysates were then harvested with radioimmunoprecipitation assay (RIPA) buffer (Thermo Fisher Scientific) containing protease inhibitor (Roche Molecular Biochemicals; Indianapolis, IN, USA). The cell lysates were analyzed with a standard Western blot technique and probed with primary antibodies for poly (ADP-ribose) polymerase (PARP) (1:1000; Cell Signaling Technology, Inc., Danvers, MA, USA) and β-actin (1:500; Cell Signaling Technology, Inc.), followed by anti-rabbit and anti-mouse horseradish peroxidase (HRP)-conjugated secondary antibodies (1:3000; Cell Signaling Technology, Inc.). The blots were then stained with Opti-ECL HRP Reagent Kit (Bioman; New Taipei City, Taiwan) for visualization using the ChemiDoc Imaging System (Bio-Rad; Hercules, CA, USA). Densitometry was performed using ImageJ software (version 1.53e) developed by W. Rasband (National Institutes of Health, Bethesda, MD, USA).

### 2.5. Annexin V/Propidium Iodide Double Staining

Cells seeded in 12-well plates (2 × 10^5^ cells per well) were infected with MV-FLuc (MOI 0.1), incubated for 48 h, and treated with UA (75 µM) for another 72 h. For analysis, the cells were trypsinized, washed twice with ice-cold PBS, and resuspended in binding buffer containing 1 µL/mL propidium iodide (PI) and 1 µL/mL fluorescein isothiocyanate (FITC)-conjugated annexin V (eBioscience™ Annexin V-FITC Apoptosis Detection Kit; Thermo Fisher Scientific). Fluorescence signals were then acquired and analyzed using the Sony SA3800 Flow Cytometer (Sony Biotechnology Inc., Shanghai, China).

### 2.6. Reactive Oxygen Species (ROS) Detection by 2′,7′-Dichlorodihydrofluorescein Diacetate (H_2_DCFDA) Staining

Cells seeded in 24-well plates (5 × 10^4^ cells per well) were infected with MV-FLuc (MOI 0.1), incubated for 48 h, and treated with UA (75 µM) for another 72 h. Cells were then stained with H_2_DCFDA (20 µM; Sigma-Aldrich) diluted in DMEM for 60 min, washed twice in PBS, and resuspended in PBS. Fluorescence signals were then acquired and analyzed using the Sony SA3800 Flow Cytometer.

### 2.7. Mitochondrial Membrane Potential (MMP) Measurement

MMP was measured using the JC-10 Mitochondrial Membrane Potential Assay Kit (Abcam; Cambridge, UK). Cells seeded in 24-well plates (5 × 10^4^ cells per well) were infected with MV-FLuc (MOI 0.1), incubated for 48 h, and treated with UA (75 µM) for another 24 h. After treatment, the cells were trypsinized, washed with PBS, and incubated in 200 µL of JC10 loading dye for 30 min at room temperature. Fluorescence signals were then acquired and analyzed using the Sony SA3800 Flow Cytometer.

### 2.8. Statistical Analysis

Data are expressed as means ± standard deviation (SD) from three independent experiments. Statistical significance was determined using Student’s *t*-test or one-way analysis of variance (ANOVA) with a post hoc multiple comparisons test in GraphPad Prism software (version 9.0.2). *p* < 0.05 was considered statistically significant.

## 3. Results

### 3.1. Cytotoxicity of UA and Oncolytic MV on HCC Cells

**Figure 1:**
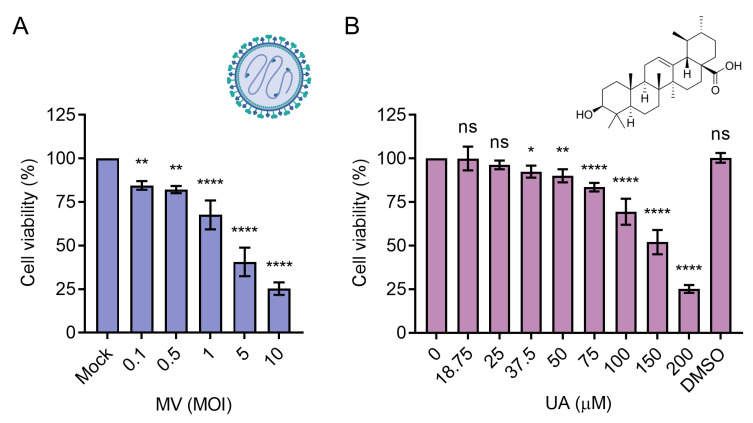
Cell viability of Huh-7 cells treated with oncolytic measles virus (MV) or ursolic acid (UA) was measured using Cell Counting Kit-8 (CCK-8). (**A**) Cells were infected with MV at multiplicity of infection (MOI) 0.1–10 for 1.5 h, washed with PBS, and further incubated for 120 h before cell viability was measured. (**B**) Cells were treated with UA (18.75–200 µM) for 72 h before cell viability was measured. DMSO (0.5%) served as a solvent control for UA. Data shown are mean ± SD from three independent experiments. One-way ANOVA with post hoc multiple comparisons test was performed (* *p* ≤ 0.05, ** *p* ≤ 0.01, **** *p* ≤ 0.0001, ns = non-significant). Schematic of MV created with BioRender.com.

We first determined the individual cytotoxicity of UA and oncolytic MV on Huh-7 HCC cells, which are permissive to MV infection [27]. UA or oncolytic MV was prepared in various concentrations or viral multiplicities of infection (MOIs) and added to Huh-7 cells. At 120 h postinfection (hpi), the viability of Huh-7 cells infected with low-dose MV was 80–85% (Figure 1A). MV at MOI 1 decreased cell viability to 67%, and MV at MOIs 5 and 10 further reduced cell survival to 40% and 25%, respectively (Figure 1A). UA (Figure 1B) also showed a dose-dependent cytotoxicity on Huh-7 cells after 72 h of incubation. Cell viability was over 90% at concentrations below 50 µM (Figure 1B). UA treatment at 100 µM and 200 µM decreased Huh-7 viability to 63% and 25%, respectively (Figure 1B). The 50% cytotoxic concentration (CC_50_) value was estimated to be MOI 2.75 for MV and 140.1 µM for UA. MV MOIs and UA concentrations below CC_50_ values were then selected for further synergistic analysis.

### 3.2. Combinations of MV and UA Treatment Produce Synergistic Killing Effect

**Figure 2:**
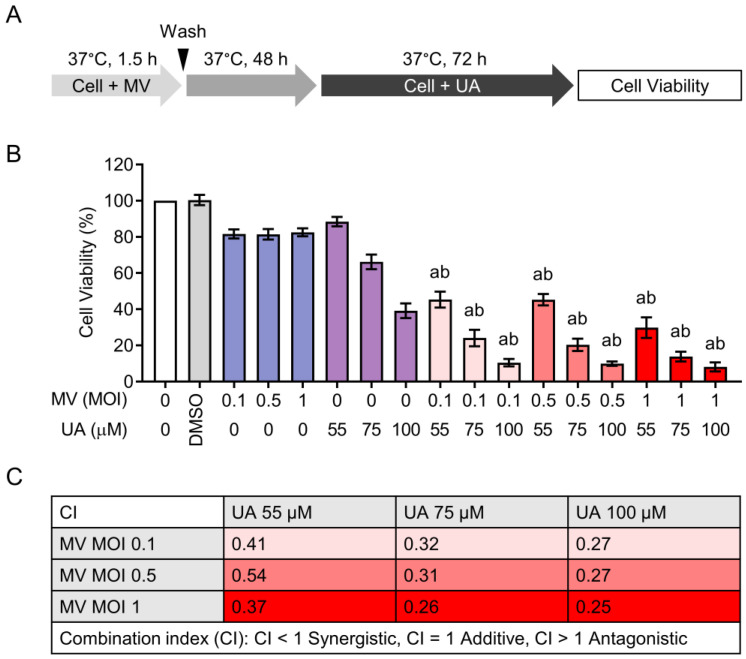
Combination treatment of MV and UA on Huh-7 cells. (**A**) Schematics of treatment schedule and cell viability measurement. (**B**) Huh-7 cells were infected with MV (MOI 0.1, 0.5, or 1), and after 48 h incubation, cells were subsequently treated with UA (55, 75, or 100 µM) for another 72 h. Cell viability was measured using CCK-8. (**C**) Combination index (CI) of each MV and UA combination from (**B**) was calculated using Chou–Talalay method. All data shown are mean ± SD from three independent experiments. One-way ANOVA with post hoc multiple comparisons test was performed to compare the difference between each combination and MV alone (a: *p* ≤ 0.05) or UA alone (b: *p* ≤ 0.05) at the respective MOI or drug concentration.

To determine the synergistic effect of MV and UA, Huh-7 cells were first infected with MV (MOI 0.1, 0.5, or 1), incubated for 48 h, and then treated with UA (55, 75, or 100 µM) for 72 h before cell viability was measured using the CCK-8 (Figure 2A). All combinations significantly decreased cell survival compared to MV alone and UA alone treatments, resulting in 4–45% cell viability (Figure 2B). We further evaluated the combination index (CI) of each MV and UA combination to see if MV and UA worked in synergy based on the Chou–Talalay method [26]. The computed CI values were all below 1 (Figure 2C), which indicates synergistic activity between the two agents. These observations showed that MV and UA combinations could synergistically increase cell death in Huh-7 cells.

### 3.3. Combination Treatment of UA and MV Promotes Apoptosis due to Increased Oxidative Stress and Mitochondrial Damage

**Figure 3:**
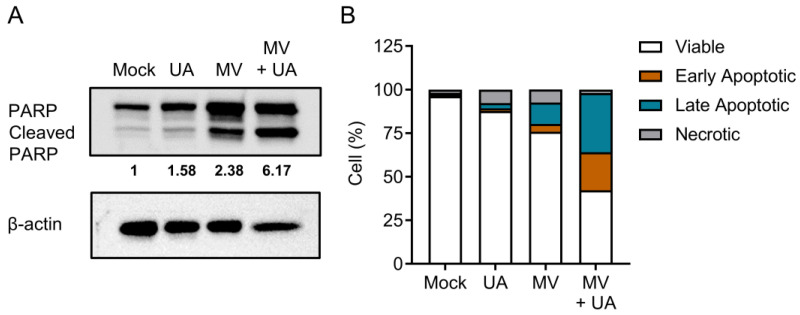
Apoptosis analyses of Huh-7 cells treated with MV and UA. Cells were infected with MV (MOI 0.1), incubated for 48 h, and treated with UA (75 µM) for another 72 h. (**A**) Whole cell lysates were harvested for Western blot analysis of poly (ADP-ribose) polymerase (PARP) cleavage. Density of cleaved PARP was first normalized to β-actin and then normalized to mock treatment. Representative data from three independent experiments are shown. (**B**) Cells were collected and stained with propidium iodide (PI) and annexin V conjugated with fluorescein isothiocyanate (FITC) for analysis of apoptosis induction by flow cytometry. Means from three independent experiments are shown.

Since MV and UA alone both induce apoptosis [28,29], we anticipated that apoptosis would be enhanced in cells treated with MV and UA combined. We first examined the level of poly (ADP-ribose) polymerase (PARP) cleavage, an apoptosis marker [30], in the MV- and/or UA-treated Huh-7 cells. Indeed, PARP cleavage was enhanced in cells treated with the combination of MV and UA (Figure 3A). We further performed a flow cytometry-based annexin V/propidium iodide (PI) staining and confirmed that both early and late apoptotic cell populations were increased in cells treated with the combination of MV and UA (Figure 3B). These results suggested that the MV + UA combination led to more cell death through apoptosis.

**Figure 4:**
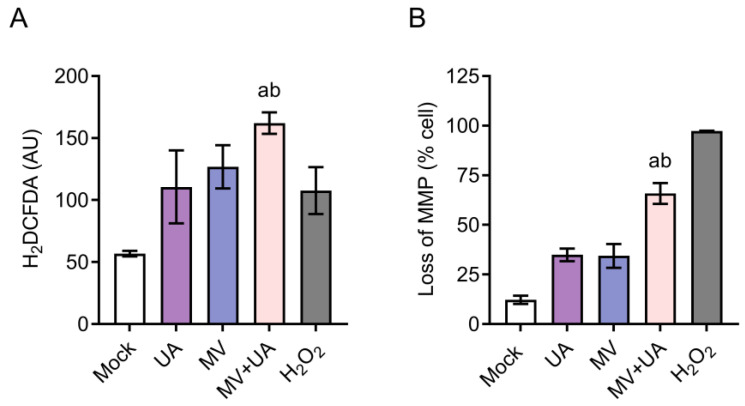
Detection of reactive oxygen species (ROS) stress and loss of mitochondrial membrane potential (MMP) in cells treated with MV and UA. (**A**) Induction of ROS stress was measured by 2′,7′-dichlorodihydrofluorescein diacetate (H_2_DCFDA) staining. Cells were infected with MV (MOI 0.1), incubated for 48 h, and treated with UA (75 µM) for another 72 h. Treated cells were then incubated with H_2_DCFDA (20 µM) for 60 min and analyzed by flow cytometry. H_2_O_2_ treatment (10 mM) for 60 min served as a positive control. AU: Arbitrary unit. (**B**) Reduction of MMP was measured by the JC-10 Mitochondrial Membrane Potential Assay Kit. Cells were infected with MV (MOI 0.1), incubated for 48 h, and treated with UA (75 µM) for another 24 h. Treated cells were then incubated with JC-10 staining buffer for 30 min and analyzed by flow cytometry. H_2_O_2_ treatment (200 μM) for 60 min served as a positive control. Data shown are mean ± SD from three independent experiments. Student’s *t*-test was performed to compare the difference between the MV + UA combination and MV alone (a ≤ 0.05) or UA alone (b ≤ 0.05).

To further investigate the mechanism of enhanced apoptosis induced by this combination, we next assessed the oxidative stress level and mitochondrial membrane potential (MMP), since UA is known to exert its anti-cancer effect through targeting the mitochondrial pathway in cancer cells [29]. Reactive oxygen species (ROS) production was detected using 2′,7′-dichlorodihydrofluorescein diacetate (H_2_DCFDA) staining. The MV + UA combination treatment induced a higher level of ROS than either treatment alone (Figure 4A). In addition, we also saw an increased proportion of cells treated with MV + UA losing their MMP (Figure 4B). These observations suggested that MV + UA could promote ROS accumulation, causing oxidative stress and destabilization of MMP, which ultimately led to increased apoptosis.

### 3.4. Synergistic Cytotoxicity of Oncolytic MV and UA Combination on HCC Cells Harboring Replicating Hepatitis B Virus (HBV) or Hepatitis C Virus (HCV)

**Figure 5:**
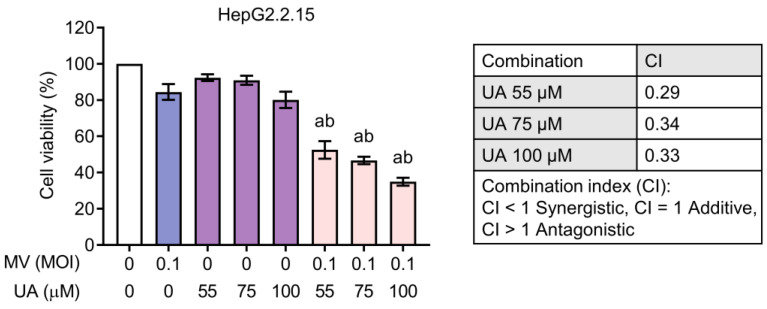
Combination treatment of MV and UA on HepG2.2.15 cells. Cells were infected with MV (MOI 0.1), and after 48 h incubation, cells were subsequently treated with UA (55, 75, or 100 µM) for another 72 h. Cell viability was measured using CCK-8. Data shown are mean ± SD from three independent experiments. One-way ANOVA with post hoc multiple comparisons test was performed to compare the difference between each combination and MV alone (a ≤ 0.05) or UA alone (b ≤ 0.05). Combination index (CI) of each MV and UA combination was calculated using Chou–Talalay method.

**Figure 6:**
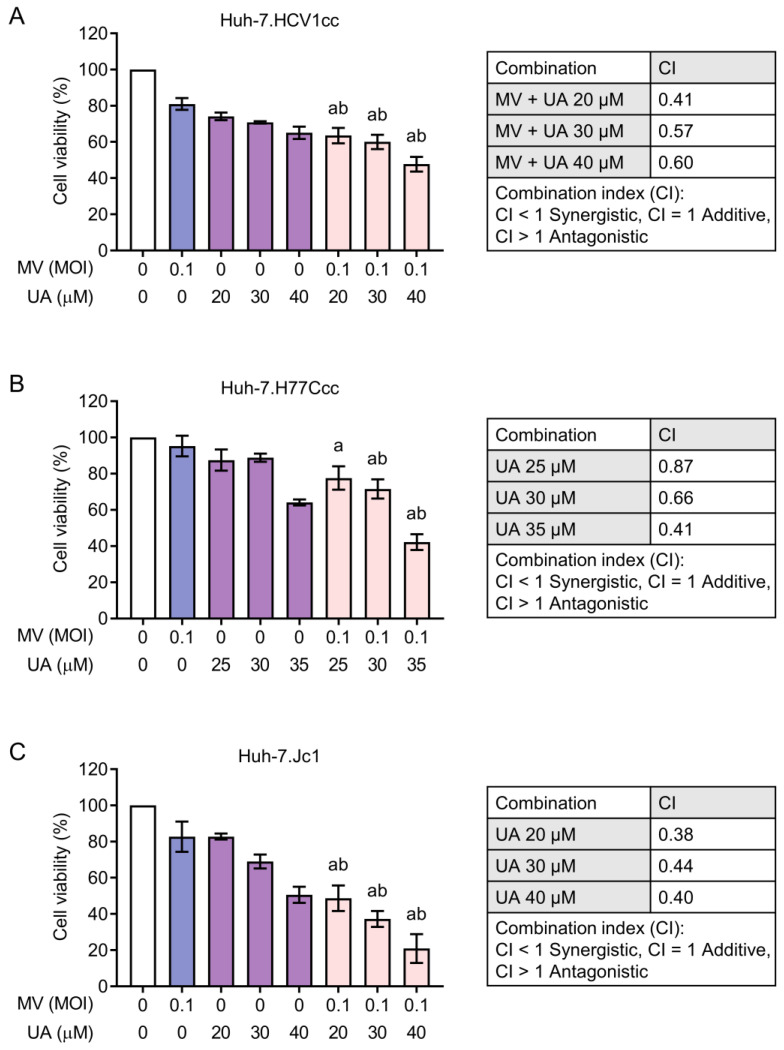
Combination treatment of MV and UA on HCV-expressing (**A**) Huh-7.HCV1cc, (**B**) Huh-7.H77Ccc, and (**C**) Huh-7.Jc1 cells. Cells were infected with MV, and after 48 h incubation, cells were subsequently treated with UA for another 72 h. Cell viability was measured using CCK-8. Data shown are mean ± SD from three independent experiments. One-way ANOVA with post hoc multiple comparisons test was performed to compare the difference between each combination and MV alone (a ≤ 0.05) or UA alone (b ≤ 0.05). Combination index (CI) of each MV and UA combination was calculated using Chou–Talalay method.

Since HBV and HCV are two major etiologies of HCC, we next examined whether the MV + UA combination could also exert synergistic toxicity on HCC cells stably established with HBV or HCV full-length replicating genomes. HepG2.2.15 contains cloned HBV DNA and produces intact HBV particles [31]. For the HCV particle-releasing cells, Huh-7.HCV1cc and Huh-7.H77Ccc both contain a genotype 1a genome [23], and Huh-7.Jc1 contains a genotype 2a genome [24]. As shown in Figure 5, the MV + UA combination treatment similarly produced synergistic cytotoxicity in HepG2.2.15 cells, resulting in 48–66% cell death. On the other hand, the HCV-expressing cells appeared more sensitive to UA compared to the parental Huh-7 cells (Figure 6). The MV + UA combination treatment also synergistically reduced cell viability, killing up to 53%, 58%, and 80% of Huh-7.HCV1cc, Huh-7.H77Ccc, and Huh-7.Jc1 cells, respectively. Altogether, these results demonstrated that HCC cells containing replicating viral genomes are susceptible to synergistic killing induced by the MV + UA combination.

## 4. Discussion

UA has demonstrated its anti-cancer effect in several cancer types including breast cancer, colorectal cancer, ovarian cancer, lung cancer, and prostate cancer by targeting multiple signaling pathways such as MAPK, NF-κB, JAK/STAT, PI3K/Akt/mTOR, and Wnt/β-catenin [32]. In HCC, previous studies have shown that UA induces apoptosis through both extrinsic and intrinsic pathways [17,33]. UA has also been suggested to inhibit proliferation signaling pathways including Ras/MAPK, PI3K/Akt, and STAT3 [19,20,33], downregulate the apoptosis inhibitor X-linked inhibitor of apoptotic protein (XIAP) [15], and suppress the transcription factor Sp1 [18] (which is a poor prognostic factor for many cancers [34]). Our present study showed that the combination treatment of UA and oncolytic MV synergistically enhanced Huh-7 cell death (Figure 2) through enhanced apoptosis, as demonstrated by the accumulation of cleaved PARP and annexin V-positive cells (Figure 3). We also found increased ROS level and reduced MMP in cells treated with the MV + UA combination (Figure 4), indicating that the combination may induce apoptosis through the mitochondrial pathway. Although UA has been suggested to reduce ROS production in brain disease and kidney disease animal models [35], increased ROS levels were, however, reported in esophageal [36] and intestinal cancer cells [37] treated with UA, as well as osteosarcoma cells treated with UA and zoledronic acid [38]. This difference may be due to the altered redox environment in cancer cells, which makes cancer cells potentially more susceptible to oxidative stress-induced cell death [39].

In addition, we observed that the combination of MV and UA similarly exerted synergistic effect on HBV- or HCV-expressing cells (Figure 5 and Figure 6). This is of therapeutic importance as HCV and HBV infections are two major etiologies of HCC, and the timing of treating viral hepatitis may occur at the time of HCC diagnosis (recommended for hepatitis B) or after complete treatment of HCC (recommended for hepatitis C) [40]. UA has been proposed to inhibit HBx-mediated tumorigenic effects such as matrix metalloproteinase 3 secretion and anti-apoptotic signaling [41]. UA could also inhibit the activity of HCV NS5B RNA-dependent RNA polymerase [42]. Our results revealed that both HBV- and HCV-expressing cells were susceptible to the synergistic cytotoxicity of MV + UA, and the HCV-expressing cells appeared more sensitive to UA treatment (Figure 5 and Figure 6). These results indicate the possibility of using the MV + UA combination as a treatment modality in HBV- or HCV-infected HCC.

Although oncolytic virotherapy is a novel strategy against cancer, its development has been met with some challenges, including limited efficacy while requiring high dosages of the virus. Combination therapy with other anti-cancer drugs/treatments has been a popular choice to boost the efficacy of OVs [43,44,45], as they can operate with distinct mechanisms mutually exclusive to the OVs, thereby synergistically potentiating the tumor oncolysis while lowering the viral dose necessary to achieve response. Examples of combinations for MV-based oncolytic virotherapy include radiotherapy [46], camptothecin [47], gemcitabine [48,49], paclitaxel [50], anti-epidermal growth factor receptor (EGFR) monoclonal antibody nimotuzumab [51], temozolomide and radiotherapy [52], and small molecules such as baicalein and cinnamaldehyde [11], heat shock protein inhibitors [53], and HDAC inhibitor resminostat [10,54]. Our current discovery that UA can boost MV’s oncolytic potency against HCC adds to the growing list of small molecules that can be combined with oncolytic MV. Additionally, we have previously observed that UA can also boost MV’s oncolytic effect in breast cancer cells [12]. Thus, UA may be a versatile anti-cancer agent that can be incorporated in combinations with oncolytic MV for treatments against various cancer types and merits further exploration in this avenue. Treatment schedules other than the currently used “viral sensitization” approach (i.e., establish virus infection first followed by drug treatment), including “drug sensitization” (drug treatment before virus infection) and co-treatment approaches, may be further explored in future studies to design an optimized schedule. Finally, the investigation of UA treatment in several clinical trials (e.g., NCT02337933, NCT05776862, NCT02401113) also highlights its potential for further clinical development, including with oncolytic virotherapy.

Collectively, in the current study we have shown that the MV + UA combination could synergistically cause cancer cell death in Huh-7 cells with enhanced apoptosis mediated through the mitochondrial pathway. Moreover, HCC cells containing replicating full-length HBV or HCV genomes were also susceptible to the combination treatment with synergistic effect. These results suggest that the MV + UA combination merits further investigation and development as a strategy for HCC management.

## Data Availability

Data are contained within the article.
